# Whole-Genome Sequences of Two Arthrobacter Strains Isolated from a Holm Oak Rhizosphere Affected by Wildfire

**DOI:** 10.1128/genomeA.00071-18

**Published:** 2018-03-15

**Authors:** Antonio J. Fernández-González, Ana V. Lasa, Manuel Fernández-López

**Affiliations:** a Departamento de Microbiología del Suelo y Sistemas Simbióticos, Estación Experimental del Zaidín, Consejo Superior de Investigaciones Científicas, Granada, Spain

## Abstract

We report here the draft genome sequences of two Arthrobacter strains isolated from a holm oak forest affected by wildfire. Both strains were shown to act as plant growth promoters, with AFG20 being a member of the most abundant group found in this soil and AFG7.2 being the strain with the highest indole-3-acetic acid production level.

## GENOME ANNOUNCEMENT

In a previous study, a collection of Arthrobacter strains was isolated from the burned rhizosphere of holm oaks (1). Sampling was done 30 months after a wildfire that affected 3,426.74 ha in Sierra Nevada National Park, Spain (2). Phenotypic assays in different media and *in planta* were performed to study their plant growth-promoting abilities (plant growth-promoting rhizobia [PGPR]) (3) for consideration as bioinoculants for burned forest recovery. Arthrobacter spp. have also been found to degrade a wide variety of compounds, including aromatic molecules, organochloride, pesticides, etc. (4). In order to deepen the knowledge of the genes involved in the adaptation of this genus under the severe conditions resulting from a forest fire and its potential as PGPR, the genomes of 2 strains have been sequenced. The strains chosen were AFG7.2, because of its high indole-3-acetic acid (a phytohormone produced by some bacteria [5]) production levels, and AFG20, because it was the only strain from the main group, which is related to Arthrobacter globiformis.

Genomic DNA was isolated using the RealPure genomic DNA extraction kit (Durviz, Valencia, Spain), with the following modifications to improve the cellular lysis: keep the centrifuged bacterial culture at −20°C for 24 h and incubate it twice with lysozyme. Whole-genome sequencing was performed using the Kapa DNA library preparation kit, using 100-bp paired-end sequencing by Illumina. Sequence trimming was carried out in CLC Genomics Workbench 9.0.1. *De novo* assemblies were performed using the Geneious 11.0.3 assembler with medium-low sensitivity. A total of 9,287 contigs were constructed for the isolate AFG7.2 and 7,258 contigs for AFG20. Only contigs that were bigger than 500 bp, mapped by more than 1,000 reads with concordance in the BLASTn best hit and in the mean coverage of each column (MCC), were retained (Table 1). In AFG7.2, contig 17 has an MCC of 1,006×. Since this contig belongs to the ribosomal genes and considering its MCC value, we expect to have 4 copies of this operon, with 2 of them in tandem, with a gene coding for a β-d-galactosidase between. On the other hand, in AFG20, we found 5 contigs with highly different MCC and annotated as plasmids. Particularly, contig 1 was manually cut into two contigs, 1 right and 1 left. The contig 1 left fragment contained the rRNA operon at the end of the sequence, and considering the MCC of this region, we expect this genome to have between 7 and 8 copies of this operon.

**TABLE 1:** Assembly information and GenBank accession numbers of the two Arthrobacter strains

Strain	No. of reads	Final no. of contigs	Contig lengths (nt)				*N*_50_ (nt)	GC content (%)	MCC (×)	Accession no.
			Total	Minimum	Maximum	Avg				
AFG7.2	16,073,554	26	4,049,802	635	765,007	176,079	411,457	64.9	314–484	PJMB00000000
AFG20	20,542,360	49	4,908,550	524	444,696	100,174	222,991	65.9	324–467	PJMC00000000

At this point, it is important to warn others to perform at least a BLASTn search on the resulting contigs to remove from the GenBank database genome projects some contaminant contigs/scaffolds coming from Illumina Library constructions. We observed this with the case of the complete genome of bacteriophage PhiX174, which was an ∼5-kb contig not only present in our assemblies but also in prokaryotic genome projects, like for Escherichia coli FHI92 (GenBank accession number LM997153), and in eukaryotic genome projects, like for Taenia asiatica (GenBank accession number LM128679), among others.

### Accession number(s).

These Arthrobacter genomes are publicly available at the GenBank database (see Table 1 for accession numbers).
